# Rationally Designed Variants of α-Synuclein Illuminate Its *in vivo* Structural Properties in Health and Disease

**DOI:** 10.3389/fnins.2018.00623

**Published:** 2018-09-25

**Authors:** Ulf Dettmer

**Affiliations:** Ann Romney Center for Neurologic Diseases, Department of Neurology, Brigham and Women’s Hospital, Harvard Medical School, Boston, MA, United States

**Keywords:** α-synuclein, structure, proteotoxicity, mutagenesis, multimerization, Parkinson’s disease

## Abstract

α-Synuclein (αS) is a conserved and abundant neuronal protein with unusual structural properties. It appears to partition between folded and unstructured states as well as between membrane-bound and aqueously soluble states. In addition, a switch between monomeric and tetrameric/multimeric states has been observed recently. The precise composition, localization and abundance of the multimeric species are under study and remain unsettled. Yet to interfere with disease pathogenesis, we must dissect how small changes in αS homeostasis may give rise to Parkinson’s disease (PD), dementia with Lewy bodies (DLB) and other human synucleinopathies. Rationally designed αS point mutations that prevent the protein from populating all states within its normal folding repertoire have continued to be instrumental in bringing new insights into its biochemistry *in vivo*. This review summarizes biochemical and cell biological findings about αS homeostasis from different labs, with a special emphasis on intact-cell approaches that may preserve the complex, metastable native states of αS.

## αS in Health and Disease

“Synucleinopathies" comprise Parkinson’s disease (PD), dementia with Lewy bodies (DLB), multiple system atrophy and also Alzheimer’s disease. DLB is the most common cause of dementia after AD and vascular dementia (Meeus et al., 2012), while PD is the most common neurodegenerative disease after AD, with an estimated life-time risk of 1.5% globally (Tanner and Goldman, 1996). Typical pathology of synucleinopathies comprises neuronal loss and neuronal/neuritic aggregation of α-synuclein (αS), a protein of 140 amino acids (aa) with an incompletely defined function involving synaptic vesicle trafficking (e.g., Scott and Roy, 2012; Vargas et al., 2014; Wang et al., 2014). Since its discovery as the first causative gene product for PD (Polymeropoulos et al., 1997) and the major constituent of Lewy bodies and Lewy neurites (large aggregate structures) in patients’ brains (Spillantini et al., 1997), αS has been increasingly implicated as a key pathogenic protein in sporadic and familial PD (fPD). αS missense mutations, copy number variants, and upregulated expression have each been associated with fPD (Polymeropoulos et al., 1997; Krüger et al., 1998; Singleton et al., 2003; Zarranz et al., 2004; Fuchs et al., 2008; Appel-Cresswell et al., 2013; Kiely et al., 2013; Lesage et al., 2013; Proukakis et al., 2013; Pasanen et al., 2014) or an fPD/DLB spectrum [especially E46K (Zarranz et al., 2004)]. Consequently, both native and altered αS folding states are of great interest as regards normal biology and the mechanisms, diagnostics and disease-modifying therapeutics of synucleinopathies. In order to prevent a pathological state of any protein, it is of central importance to understand its physiological state in all details. Nonetheless, αS structure and precisely how it is altered in the synucleinopathies remain unclear. A well-recognized αS species *in vitro* is the soluble unfolded monomer (Weinreb et al., 1996), and a recent publication showed that this state can persist in considerable part when exogenous unfolded recombinant monomers are delivered into cultured mammalian cells by electroporation (Theillet et al., 2016). However, this in-cell liquid phase NMR analysis did not rule out the existence of other αS species, as the method is unable to detect membrane-bound or multimeric αS forms (Alderson and Bax, 2016). At least 10% of cellular αS is found in membrane fractions (Kahle et al., 2000; Fortin et al., 2010; Dettmer et al., 2015a), and transiently membrane-associated αS has been characterized as monomeric and helically folded (George et al., 1995) or multimeric and (presumably) folded (Burré et al., 2014). In addition, previous (Bartels et al., 2011) and recent (Iljina et al., 2016) studies report soluble, multimeric αS forms that have α-helical conformation, resist aggregation, and are distinct from pathological, β-sheet-rich oligomers that are the hallmark of synucleinopathies. If all αS forms that have been repeatedly described are relevant, they likely exist in a dynamic equilibrium with each other. Perturbation of the neuronal αS equilibrium in neurons on the other hand, may be the starting point for pathological αS insolubility and misfolding. Since their discovery, the known fPD/DLB-linked αS missense mutations have been obvious candidates for studying perturbed αS equilibria, but rationally designed variants have been informative as well.

## The Repetitive Structure of αS

**FIGURE 1:**
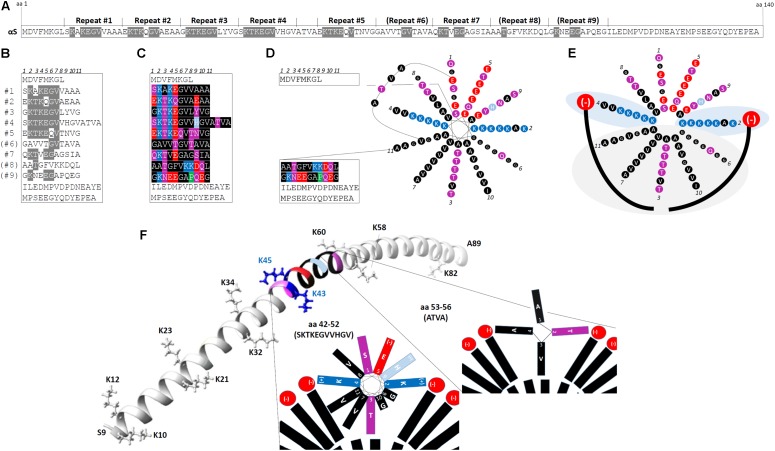
The repetitive structure of αS. **(A)** The linear 140-aa sequence of human wt αS. 11-aa repeats 1–9 with the core consensus motif “KTKEGV” are indicated, poorly conserved repeats are in brackets; aa that fully conform to “KTKEGV” are highlighted in gray. **(B)** Schematic of wt human αS by aligning its aa sequence via the KTKEGV motifs; aa that fully conform to “KTKEGV” are highlighted in gray. Repeats 1–9 are interrupted only once: by “ATVA” between repeat 4 and 5. **(C)** Color-coded schematic of wt human αS by aligning its aa sequence via the KTKEGV motifs. Blue indicates basic (light blue: histidine), red: acidic, purple: polar uncharged, and black: non-polar residues. In addition to KTKEGV, the polar character of positions 1 and 9 as well as the non-polar, hydrophobic character of positions 8, 10, and 11 are relatively well conserved. **(D)** Color-coded schematic of wt human αS with its 11-aa repeats 1–7 arranged in the helical wheel (11/3 helix: 3 turns over 11 residues) of a membrane-induced amphipathic helix. Blue indicates basic (light blue: histidine), red: acidic, purple: polar uncharged, and black: non-polar residues. The design of the wheel diagram has been adapted from Bendor et al. (2013). **(E)** Color-coded schematic of αS repeats 1-7 (omitting “ATVA” between repeats 4 and 5) in an 11/3 helical wheel, embedded in the outer leaflet of a curved vesicle membrane (negatively charged lipid head-groups in red, fatty acid ‘tails’ in black). The helix is stabilized by hydrophobic interactions (gray area) and electrostatic interactions (blue area). **(F)** Position of repeat 4 (aa 42–52) and ATVA (aa 53–56) within the extended 11/3 helix of αS (only aa 9–89 are shown). The positions of the lysine “wings” are indicated (the hydrophobic half of the helix is below, the hydrophilic half above the plane that is defined by the lysines). The structure is based on data by Jao et al. (2008) (courtesy Ralf Langen lab, USC).

The N-terminal two thirds of αS contain up to nine 11-residue imperfect repeats, with the consensus core motif being KTKEGV (Bendor et al., 2013). **Figure 1A** highlights the KTKEGV motifs within the 140 aa sequence, and **Figure 1B** displays the αS aa sequence after aligning it by the KTKEGV motifs. Repeats 1–5 and 7 are highly conserved, repeat 9 is partially conserved and repeats 6 and 8 are poorly conserved. The repeats are interrupted by 4 aa (ATVA) between repeats 4 and 5. In addition to the core motif (KTKEGV) that encompasses positions 2–7 of the 11-aa repeat, the polar character of positions 1 and 9 as well as the non-polar, hydrophobic character of positions 8, 10, and 11 are relatively well conserved, as visualized by the color-code in **Figure 1C** (see legend). The repeats are highly conserved, both across vertebrate species and among the three homologs α-, β- and γ-synuclein. The KTKEGV motif has not been observed in non-vertebrates, and no similar sequence has been identified outside the synuclein protein family. However, perilipins (Londos et al., 1999) as well as apolipoproteins and certain plant proteins (George et al., 1995) exhibit a similar overall structure, i.e., they contain 11-aa repeats with a similar pattern of charged/polar and hydrophobic aa. Like αS, apolipoproteins interact with lipid membranes via their N-terminal regions and, interestingly, they are the protein class that is over-represented in amyloid diseases: so far four apolipoproteins, SAA, Apo AI, Apo AII, and Apo AIV, have been described in the context of amyloidosis (Sipe et al., 2014). The 11-aa repeats enable such polypeptides to form amphipathic 11/3 helices at membranes (different from true α-helices): after exactly three turns, position 1 of the next 11-aa repeat is reached. **Figure 1D** illustrates in a simplified fashion the 11/3 helix formation of αS for repeats 1–7 in the context of the rest of the protein including the ATVA intervening sequence. The formation of helical αS on membranes was predicted from its sequence and formally demonstrated by binding to artificial membranes *in vitro* (Davidson et al., 1998). In a simplified diagram that focuses on repeats 1–7 and ignores ATVA, **Figure 1E** depicts two forces that attract amphipathic αS helices to cellular membranes: (i) light gray area: hydrophobic interactions between fatty acyl chains (long curved black lines in **Figure 1E**) and the hydrophobic half of the αS amphipathic helix; and (ii) light blue areas: electrostatic interactions between positively charged lysines (K) on opposite sides of the helix and negatively charged membrane lipid headgroups (e.g., phosphatidylserine, phosphatidic acid, phosphatidylinositol headgroups; depicted in red) (Zhu and Fink, 2003). While a “bent” α-helix was detected on small-diameter micelles by nuclear magnetic resonance (Eliezer et al., 2001; Ulmer and Bax, 2005), the extended 11/3 helix was observed for spin-labeled protein on artificial membranes, which have a larger diameter than micelles and are expected to model the *in vivo* conformation better (**Figure 1F**: aa 9–89) (Jao et al., 2004, 2008). Thus, through the membrane interaction of multiple KTKEGV repeats, αS likely forms one long 11/3 helix that lies along the outside surface of cellular vesicle membranes, at least half-buried in the bilayer (Bussell et al., 2005; Jao et al., 2008; Wietek et al., 2013), while the C-terminal ∼30–40 aa remain unfolded. Moreover, the preference of αS for curved membranes (cellular vesicles) instead of relatively flat membranes (ER or plasma membrane) is well established (Middleton and Rhoades, 2010; Jensen et al., 2011). **Figure 1F** illustrates the positions of repeat 4 and ATVA (both color-coded) within the extended 11/3 helix of αS (only aa 9–89 are shown). Moreover, the positions of the lysine “wings” are shown (the hydrophobic half of the helix is below, the hydrophilic half above the plane that is defined by the lysines).

Importantly, purified recombinant αS in solution behaves like a natively unfolded protein *in vitro* (Weinreb et al., 1996; Bertoncini et al., 2005). The *in vivo* occurrence of this αS conformation, presumably in addition to membrane-associated helical monomers, has recently been suggested by intact-cell NMR (Binolfi et al., 2012; Theillet et al., 2016). In addition to those monomeric states, native multimeric αS assemblies have been observed by several groups (Bartels et al., 2011; Wang et al., 2011, 2014; Dettmer et al., 2013; Gurry et al., 2013; Westphal and Chandra, 2013; Burré et al., 2014; Gould et al., 2014; Iljina et al., 2016). The characterization of the multimeric αS species ranges from soluble tetramers (Bartels et al., 2011; Wang et al., 2011) to membrane-associated octamers (Burré et al., 2014). The relationship and relative abundance of all these species may be highly dependent on biological context and therefore difficult to predict from *in vitro* experiments.

**FIGURE 2:**
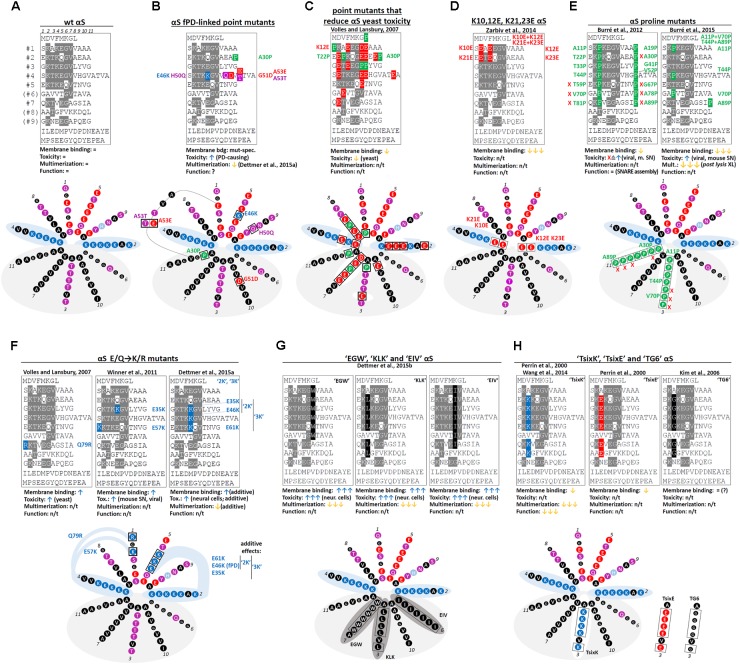
Wt αS, fPD-linked and strategic αS mutations. **(A)** Schematic of wt human αS by aligning its aa sequence via the KTKEGV motifs; aa that fully conform to “KTKEGV” are highlighted in gray (top). Color-coded (see **Figure 1**) schematic of repeats 1–7 (omitting “ATVA” between repeats 4 and 5) in an 11/3 helical wheel (bottom). **(B)** fPD-linked αS missense mutations (“ATVA” was included to illustrate A53T and A53E). **(C)** Non-toxic αS point mutations (in aa 1–89) identified by expression in yeast (most were identified in compound mutants and the focus is on E and P mutations). E at aa positions 3, 7, and 10 of each repeat destabilizes the hydrophobic interaction between αS and lipid tails, E at positions 2 and 4 destabilizes electrostatic interactions with lipid headgroups; the effect of E at position 8 is less clear. P destabilizes the helix independent of its position. **(D)** K10,12E and K21,23E. E substitutions at positions 2 and 4 of each repeat destabilize the electrostatic interaction with lipid headgroups. **(E)** Engineered αS single P mutants (left panel) and compound mutants A11P/V70P and T44P/A89P (right panel). The P substitutions reduce helix formation, thereby increasing the pool of cytosolic unfolded αS. “X” marks toxic single point mutants. **(F)** E/Q-to-K/R mutants. The positive charge of R or K presumably stabilizes the electrostatic interaction with negatively charged headgroups analogous to fPD-linked αS E46K. Left panel: yeast-toxic αS Q79R. Middle panel: E35K and E57K. Right panel: αS “2K” (E35K + E46K) and αS “3K” (E35K + E46K + E61K) amplify/exaggerate E46K. **(G)** Hydrophobic KTKEGV repeat motif mutants. Left: “EGW” (consensus motif: KTKEGW). The bulky, non-polar W instead of V at position 7 adds hydrophobicity to the amphipathic αS helix, leading to strongly increased binding. Middle: Engineered αS mutant “KLK” (KLKEGV). The non-polar L instead of T at position 3 corrects the imperfect hydrophobicity in the amphipathic helix, leading to strongly increased binding. Right: “EIV” (KTKEIV). The non-polar I instead of G at position 6 corrects the imperfect hydrophobicity in the amphipathic αS helix, leading to strongly increased binding. **(H)** KTKEGV repeat motif mutants modifying the “T” position. T at position 3 of each 11-aa repeat causes imperfect hydrophobicity in the hydrophobic face of the amphipathic αS helix, leading to only transient binding. Left: “TsixK” (KKKEGV). K instead of T at position 3 reduces the hydrophobicity in the amphipathic helix. However, the additional positive charge may lead to an aberrant binding to negatively charged lipid head groups, permitting residual, but possibly abnormal binding characterized by less helicity. Middle: “TsixE” (KEKEGV). E instead of T at position 3 reduces the hydrophobicity in the amphipathic helix. Right: “TG6” (KGKEGV). G instead of T at position 3 may have a helix-destabilizing effect, while membrane-binding might be intact. (n/t, not tested; XL, crosslinking; gray areas indicate the hydrophobic face of the amphipathic αS helix; dark gray areas: increased membrane binding/stronger helix formation; white areas: decreased membrane binding/impaired helix formation).

Over the years, several αS missense mutations have each been associated with familial PD (**Figure 2A**: wt αS, **Figure 2B**: mutants). Those are, in chronological order of publication: A53T (Polymeropoulos et al., 1997), A30P (Krüger et al., 1998), E46K (Zarranz et al., 2004), H50Q (Appel-Cresswell et al., 2013; Proukakis et al., 2013), G51D (Kiely et al., 2013; Lesage et al., 2013) and A53E (Pasanen et al., 2014). While all except A30P cluster around aa 50, their relative position in the αS amphipathic helix differs: A30P (11-aa repeat position: 11) and G51D (position 10) are in the hydrophobic half of the helix, E46K (position 5) and H50Q (position 9) are in the hydrophilic half; A53T/E are not found in the 11-aa repeat, but in the ATVA sequence between repeat 4 and 5. Neither is the effect of the substitution on the nature of the respective aa (e.g., charged vs. uncharged) unifying among them. Moreover, A30P binds to membranes less than wt (Jo et al., 2002), E46K binds more (Choi et al., 2004) and the A53T may exhibit similar binding (Bussell and Eliezer, 2004). In contrast to that, the clustering around a “putative protein loop” (Kara et al., 2013) (except A30P) and a negative impact on physiological multimer formation (Dettmer et al., 2015a) have been proposed to be a common feature of fPD-linked mutants. Strategic “exaggerations” and “analogies” of fPD-linked mutants promise to lead to a better understanding of the effect of fPD-linked mutations on αS homeostasis, especially when studied in the cellular context (see below). Many important questions in αS research are ultimately linked to understanding αS conformational homeostasis in intact cells. How are the different αS conformations linked to αS function *in vivo*? How is αS structural homeostasis maintained in health and perturbed in disease? How does αS proteinaceous aggregation start, and is that truly the key pathogenic event in human synucleinopathies? How should we design strategies for therapeutic intervention? This review will emphasize that valuable tools toward answering these questions can arise from informative point mutations that, e.g., prevent αS from populating all states within its normal folding landscape.

## αS Toxicity and Membrane Binding

About one decade after the discovery of αS as the principal Lewy body component and its initial structural characterization, Volles and Lansbury (2007) published a remarkable study on αS fibrillization and yeast toxicity. The authors screened a library of random αS point mutants both *in vitro* and in yeast in order to identify variants that could help elucidate sequence-phenotype relationships. When *in vitro* fibrillization and yeast toxicity of the αS variants were compared, no correlation of toxicity with fibrillization rate was observed, suggesting that fibrillization is not necessary for αS-induced yeast toxicity. A second screen in a library of several thousand yeast clones identified 25 non-toxic αS sequence variants. Most of these contained a mutation to either proline (P) or glutamate (E) that decreased membrane binding (**Figure 2C**) relative to wt αS (**Figure 2A**). The authors hypothesized that αS toxicity in yeast is caused by the protein binding directly to membranes at levels sufficient to non-specifically disrupt membrane homeostasis. Subsequent studies helped characterize the membrane-associated toxicity of αS in more detail: αS expression in yeast (any level of αS expression in the αS-lacking *S. cerevisiae* is “over-expression”) was found to lead to vesicle clustering/aggregation (Soper et al., 2008) and vesicle trafficking defects (Cooper et al., 2006). The relevance of these findings beyond the yeast system, and for PD pathogenesis in particular, was highlighted when similar vesicle trafficking defects were recapitulated in patient-derived αS A53T and αS triplication iPS cell cultures (Chung et al., 2013). However, in contrast to the yeast system, no pronounced toxicity was observed in the iPS cells, possibly due to lower expression levels or a better ability of mammalian cells to compensate for αS-induced membrane dyshomeostasis. While this apparent lack of immediate toxicity is consistent with PD being an insidious, relatively late-onset disease, it also suggests that a “mutation amplification” strategy might be necessary to readily detect αS-induced vesicle trafficking defects and their related toxicity in mammalian cells.

A decrease in membrane binding caused by “E mutants” was confirmed upon expression in human cells (HEK and the mesencephalic neuronal cell line MN9D) by Zarbiv et al. (2014). The authors replaced two positive lysine (K) residues with two negative glutamate (E) residues at either the first (K10,12E) or second (K21,23E) KTKEGV repeat motif (**Figure 2D**). Reduced binding of both double-point mutations relative to wt αS was determined by a quantitative phospholipid ELISA assay. In addition, the K-to-E substitutions resulted in strongly reduced levels of soluble αS oligomers, but larger intracellular inclusions. The toxicity of the mutants relative to wt αS was not addressed in the study.

The membrane binding and toxic effects of “P mutants” in neuronal cells were the subject of a comprehensive study by Burré et al. (2012). The authors generated 13 strategic αS proline mutants within seven αS KTKEGV repeats (**Figure 2E**, left panel) and analyzed them relative to wt, A30P, E46K, and A53T αS in seven assays that ranged from biochemical studies on purified αS to examining the toxicity of virally expressing αS in the mouse substantia nigra (SN) by stereotactic injections. Strikingly, proline mutations in the central region of αS, referred to as NAC (non-amyloid β component) domain (residues 61–95), as well as T59P and the fPD-linked mutations A30P, E46K, A53T increased the neurotoxicity of αS. In contrast, *all* P mutants (except G41P) both inside and outside the NAC domain significantly reduced membrane association, as the authors had expected based on the helix-breaking effects of the proline residues. In a follow-up study (Burré et al., 2015), the same group examined proline-rich αS variants A11P/V70P and T44P/A89P (**Figure 2E**, right panel), plus a quadruple mutant A11P/V70P/T44P/A89P. All these compound “P mutants” showed lack of membrane binding in various *in vitro* assays. Moreover, all compound proline mutants were significantly more toxic than wt αS in the cultured cells, and the respective P mutant-injected mice (SN, stereotactic virus injection) showed more motor deficits and more pronounced loss of tyrosine-hydroxylase-positive neurons in the SN compared to wt αS-injected mice (which themselves showed more deficits than control-injected mice). In cultured cells, transfection of A11P/V70P and T44P/A89P as well as the quadruple P mutant caused cytoplasmic inclusion formation that was significantly more pronounced than that of wt αS. While the authors did not address the nature of the inclusions in the study, they assumed that those inclusions were β-sheet-rich and thus different from the vesicle-rich inclusions that can be observed when wt αS is expressed in yeast or when αS variants with enhanced membrane binding are expressed in human cells (see below). At a first glance, these observations by Burré et al. (2015) challenged the assumption of “less membrane-binding = less (immediate) toxicity” as suggested by Volles and Lansbury (2007). However, it should be noted that (i) no proline mutations more C-terminal than V38P had been identified to be protective in yeast, consistent with the idea that NAC-domain mutations are more detrimental; (ii) αS amyloid formation has been reported to be more difficult to achieve in *S. cerevisiae* than in mammalian cells (Soper et al., 2008; Jarosz and Khurana, 2017); (iii) the only real discrepancy between the two studies are the data on A30P. The PD-causing effect of the “P mutant” αS A30P had always been at odds with the study by Volles and Lansbury (2007) and several other αS studies in yeast, where A30P behaved like a negative control for αS toxicity. In that regard, the approaches proposed by Burré et al. might overcome the previous lack of robust αS A30P cellular toxicity models.

Importantly, the study by Volles and Lansbury (2007) (**Figure 2C**) also identified the point mutation Q79R to increase αS toxicity paralleled by an increase in membrane binding (**Figure 2F**, left panel). In this context, a publication by Winner et al. (2011) is of interest, reporting the pronounced toxicity of two engineered αS variants, αS E35K and αS E57K (**Figure 2F**, middle panel), when virally expressed in rat midbrains. The overall effect of these two mutants on αS amphipathic helix formation at membranes might be similar to that of Q79R (**Figure 2F**, left panel), as additional positive charges are added to the hydrophilic half of the helix in all three cases. More importantly, these charge changes may be analogous to the effect of the fPD-linked αS E46K mutant (Zarranz et al., 2004) (**Figure 2B**). αS E46K is known to bind to membrane phospholipids more tightly than wt αS (Choi et al., 2004). It was suggested that the E46K αS amphipathic helix is more stable at phospholipid membranes due to the possibility of forming an additional salt bridge between the positively charged lysine (K) and a negatively charged phospholipid head group (Perlmutter et al., 2009). This electrostatic attraction is visualized in **Figure 2F**, and the mechanism can likely be extended to E35K and other engineered “K mutants”. Similar to E35K, the fPD-linked E46K has been reported to exhibit more pronounced toxicity than wt αS in both yeast (Lázaro et al., 2014) and mammalian cells (Íñigo-Marco et al., 2017). Furthermore, both E35K and E46K occur in the core repeat motif (KTKEGV becomes KTKKGV), and both at position 5 of the 11-aa repeat (E35K: position 5 in repeat 3; E46K: position 5 in repeat 4). This is consistent with the “dose-dependent” effects of the “2K” compound mutant E35K + E46K (**Figure 2F**, right panel) and the further extrapolation to “3K” = E35K + E46K + E61K (**Figure 2F**, right panel) (Dettmer et al., 2015a). Importantly, a “dose-dependent” increase in αS toxicity and membrane binding (PBS insolubility) was observed in mammalian cells with each additional E-to-K mutation. In several assays, transfection of the αS 3K variant into neuroblastoma cells led to strong cytotoxicity, similar to the level caused by the pro-apoptotic protein Bax (Dettmer et al., 2015a). E46K was shown to be more toxic than wt in at least one assay, while the toxicity of αS 2K was in between E46K and 3K. The pronounced cellular toxicity of αS 3K was accompanied by the development of round cytoplasmic inclusions (Dettmer et al., 2015a). Electron microscopy (EM) identified these inclusions as round, dense clusters of vesicles of different sizes that were strongly positive for αS by immunogold (Dettmer et al., 2017). In co-expression experiments, vesicular markers of various origins (endosomal, lysosomal, and Golgi), but not mitochondrial or ER membrane markers, were found to co-localize with the αS 3K inclusions (Dettmer et al., 2017). The lack of 3K overlap with ER membranes is at odds with a report on pronounced ER interactions of E46K (Mbefo et al., 2015), which will need to be consolidated in future studies. Nonetheless, the principal effects of increased toxicity apply to all E/Q-to-K/R mutations in repeat positions 1 and 5, as presented in **Figure 2F**. And this seems to be related to excess interaction with vesicular membranes and/or aberrant binding to non-vesicular membranes. Importantly, the effect of expressing αS 3K in mammalian cells resembles expressing wt human αS in yeast: (immediate) cytotoxicity, inclusion formation and vesicle clusters, linking the 3K effect to wt αS pathobiology.

While no analogy to a known fPD-linked mutant exists, another theoretical strategy of increasing αS membrane binding consists in increasing the hydrophobic interaction between αS and the fatty-acyl chains of phospholipid bilayers. The imperfect hydrophobicity of the hydrophobic half of the αS amphipathic helix (**Figure 1E**) could be enhanced by several strategies: (i) replacing the central threonine (T) of KTEKGV with an uncharged and non-polar aa such as leucine (L), changing the consensus motif to KLKEGV (abbreviated “KLK”; **Figure 2G**, middle panel); (ii) replacing the small glycine (G) with a bulkier non-polar aa such as isoleucine (I), resulting in KTKEIV (abbreviated “EIV”; **Figure 2G**, right panel); or (iii) replacing the non-polar valine (V) with a bulkier non-polar aa such as tryptophan (W), resulting in KTKEGW (abbreviated “EGW”; **Figure 2G**, left panel). Similar to αS 3K, such engineered αS mutants were enriched in membrane fractions, were immediately toxic when transfected into neuroblastoma cells, and led to round cytoplasmic inclusions that were again shown by EM and fluorescence microscopy to be clusters of vesicles of various origins (Dettmer et al., 2015b, 2017). These findings are consistent with earlier observations by Volles and Lansbury (2007), namely, that increased membrane binding and increased toxicity are correlated. A “KLK”-like αS variant termed “T6” (Pranke et al., 2011) (4 T→L and 2 T→F substitutions) has been studied *in vitro*, and the authors concluded that its strongly increased membrane binding was accompanied by a lack of specificity toward target membranes: while wt αS showed a preference for curved membranes, as observed before (e.g., Middleton and Rhoades, 2010; Jensen et al., 2011), αS T6 lost this preference and also bound to flat membranes *in vitro*. However, co-localization analyses with different cellular markers in neuroblastoma cells showed that the highly similar αS “KLK” (**Figure 2G**, middle panel) was associated with vesicular, but not ER or mitochondrial membranes, consistent with a quantitatively, but not qualitatively different binding mode (Dettmer et al., 2017).

Position 2 of the KTKEGV core motif was addressed in two additional studies. Perrin et al. (2000) characterized αS “TsixK” (KTKEGV becomes KKKEGV in six repeats; **Figure 2H**, left panel) and “TsixE” (KTKEGV becomes KEKEGV in six repeats; **Figure 2H**, middle panel). TsixK and TsixE both introduce thermodynamically unfavorable charges into the hydrophobic half of the αS amphipathic helix (TsixK: 6 lysines = 6+; TsixE: 6 glutamates = 6−). Interestingly, the authors observed pronounced membrane repulsion of TsixE, while for certain phospholipid mixtures TsixK still engaged in membrane binding despite diminished lipid-induced helicity (35% helicity instead of 70% in the lipid-bound state). The complete solubility of TsixE in the presence of membranes composed of acidic phospholipids was attributed to both electrostatic repulsion and reduced hydrophobicity in the hydrophobic face of the αS helix. A different study that involved a T substitution strategy, however, questioned to some extent the biological relevance of studying αS membranes interactions *in vitro*: Kim et al. (2006) reported that the interaction of αS with biological membranes may be quite different from that with model membranes. The authors characterized the interaction typically observed with model phospholipid membranes as spontaneous, stable and mainly driven by electrostatic attraction. In contrast, the interaction with cellular membranes was proposed to be highly dynamic and more driven by hydrophobic attraction. Kim et al. (2006) further suggested that the latter mode of αS interaction only occurs in the context of the cytoplasm and that cytoplasmic co-factors assist the interaction, consistent with findings by other researchers (Wislet-Gendebien et al., 2006; Chen et al., 2013). The authors pursued an “in-lysate” chemical crosslinking strategy and observed that cellular αS-membrane binding was characterized by the trapping of a 17 kDa membrane-bound version of αS (p17), in addition to the expected 14 kDa unmodified monomer. A “TG6” αS mutant (KTKEGV becomes KGKEGV in six repeats; **Figure 2H**, right panel), designed to break the helical structure of the protein, abolished p17 formation, while the mutant protein was apparently still detected in membrane fractions similarly to wt αS [see Figure 4 in Ref. Kim et al. (2006); note that the authors do not explicitly assess the total membrane binding of wt vs. TG6]. Analogous to “TsixK” this could indicate that for TG6 membrane binding was still possible, but helix formation was impaired.

## αS Multimerization

That cellular αS homeostasis is more complex than just a constant switching between soluble (unfolded) and membrane-associated (helical) monomers was proposed in a study by Bartels et al. (2011). First, using intact-cell crosslinking, the authors observed apparent multimeric species by Western blotting in addition to the 14 kDa monomer. Second, they used native methods to isolate cellular αS under non-denaturing conditions from various natural (non-PD) sources, including neural cells and human erythrocytes. The αS assemblies they isolated had α-helical structure by circular dichroism and were sized by two methods as ∼60 kDa tetramers that were relatively resistant to *in vitro* aggregation, compared to recombinant monomers. Subsequent work from several labs confirmed elements of this new hypothesis of the existence of physiological αS multimers in addition to free monomers and emphasized the dynamic nature of αS tetramers/multimers (Wang et al., 2011; Dettmer et al., 2013; Gurry et al., 2013; Gould et al., 2014; Luth et al., 2015). For example, chemical crosslinkers were found to trap 60 kDa αS (monomer MW: 14 kDa) in intact, living cells, but not in cell lysates (Dettmer et al., 2013). Questions regarding the specificity of crosslinked αS cellular multimers (Fauvet et al., 2012) triggered a search for defined point mutations in the molecule that would abolish αS tetramer/multimer formation, based on the rationale that identifying such mutations would argue for molecular specificity. Focusing on the semi-conserved KTKEGV core motif in the αS 11-aa repeats, Dettmer et al. (2015b) identified missense mutations that, when introduced in several repeats, led to the detection of solely 14 kDa αS monomers, with αS multimers virtually absent. Mapping the identified mutations based on the membrane-induced αS helix model revealed that most αS multimer-abolishing repeated KTKEGV motif variants (6 × KLKEGV = “KLK,” 7 × KTKEIV = “EIV,” 6 × KTKEGW = “EGW”) strongly increased the hydrophobicity of the hydrophobic half of the αS membrane-induced helix (**Figure 2G**). Another multimer-abolishing mutant (6 × KTKKGV = “KGV”; not shown) identified in the study also increased αS membrane helix formation, but likely via additional electrostatic interactions between lysine residues and lipid headgroups. 6 × KTKKGV is a further amplification of αS “3K” (3 × KTKKGV; **Figure 2F**) and, importantly, fPD-linked E46K (1 × KTKKGV; **Figures 2B,F**), which both “dose-dependently” lead to multimer abrogation and increased membrane binding (Dettmer et al., 2015a). In all cases, the increase in membrane binding and loss of αS multimerization was accompanied by inclusion formation and toxicity (Dettmer et al., 2015a,b). This raised the question of what was responsible for the observed toxicity of those particular mutants: the loss of multimerization or the increased membrane binding? If both aspects of αS biology are tightly linked, the question may be hard or impossible to answer.

The study by Dettmer et al. (2015a) also revealed that 5 different fPD-linked αS missense mutations (A30P, E46K, H50Q, G51D, and A53T) all shifted the multimer:monomer equilibrium toward more monomers, albeit not as drastically as the strategic repeated mutants. This is noteworthy because at least A30P (Jensen et al., 1998; Jo et al., 2002) and G51D (Fares et al., 2014) exhibit decreased, not increased, membrane binding, suggesting that the detrimental effect of αS monomer excess goes beyond increased membrane binding. Otherwise, not all five mutants, membrane-enriched such as E46K or cytosol-enriched such as A30P and G51D, would cause PD. The notion that αS A30P reduces both membrane binding and multimerization was supported by Westphal and Chandra (2013), and work by Burré et al. (2015) (**Figure 2E**, right panel) helped generalize the hypothesis that impaired formation of αS membrane-induced amphipathic helices causes αS monomer excess in the cytosol. According to Burré et al. (2015), the lack of membrane interaction of αS A11P/V70P and T44P/A89P monomers causes a failure to form native multimers at the membrane. These findings were consistent with αS TsixK (**Figure 2H**, left panel), which had been described to interfere with αS folding at membranes (Perrin et al., 2000), also abolishing αS multimerization, as assessed via YFP complementation on vesicle surfaces in intact neurons (Wang et al., 2014). Burré et al. (2015) concluded that reduced membrane binding results in the accumulation of monomeric, unfolded αS in the cytosol, as assessed by crosslinking in brain homogenates (but post lysis). It is plausible that an accumulation of unfolded soluble αS is the starting point of αS amyloid formation, since it is well-known that monomeric, unfolded αS can aggregate *in vitro* (Conway et al., 2000). In this context, it is important to note that in pure solutions *in vitro*, where both wt and P mutant αS are natively unfolded, αS A11P/V70P or T44P/A89P were not more aggregation-prone than wt. The P mutants aggregated faster only in the cellular context, in which wt, but not P mutant αS can interact with membranes, once again highlighting the importance of studying consequences of αS structure alterations in intact cells. However, membrane excess of αS – as observed for αS “3K,” “KLK” or “EIV” – has also been proposed to be the starting point of αS aggregation (Galvagnion et al., 2015), and the two possibilities are not mutually exclusive. Moreover, the amyloid nature of the observed inclusions in the respective studies reviewed here will require further analysis: EM revealed that “3K,” “KLK,” or “EIV” αS inclusions are primarily vesicle-rich αS inclusions and may only over time give rise to protein-rich, β-sheet αS inclusions. The exact nature of αS A11P/V70P or T44P/A89P inclusions that Burré et al. (2015) readily observed upon expressing these in cell culture has not been addressed yet.

**FIGURE 3:**
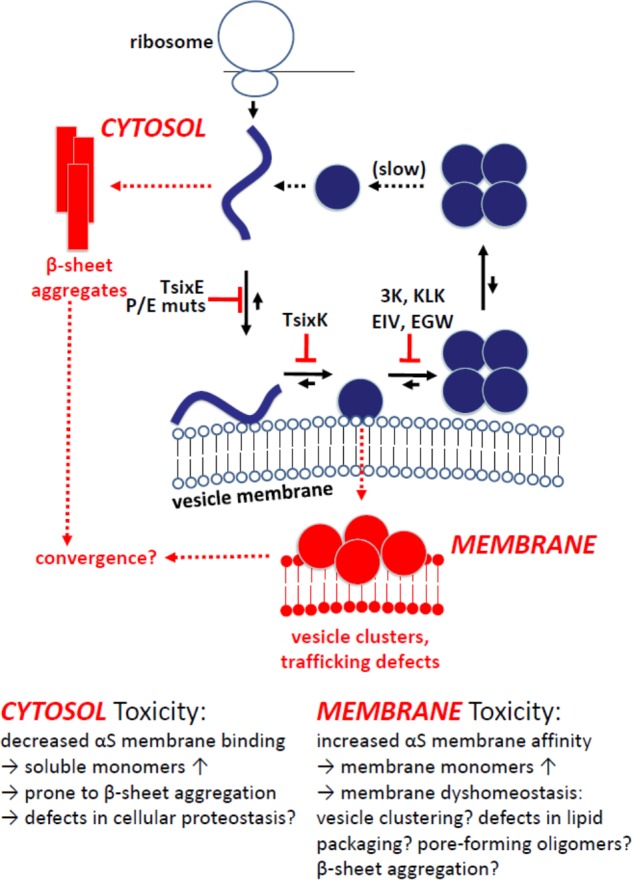
Model of cellular αS homeostasis and the effect of engineered αS mutants. Physiological situation in blue: Coming off the ribosome, αS is soluble, unfolded and monomeric. Upon binding to vesicular membranes, it adopts helical fold. Folded monomers assemble to form multimers/tetramers on membranes. Multimers/tetramers are only weakly membrane-associated and likely in an equilibrium with cytosolic multimers/tetramers. Cytosolic tetramers/multimers may have an intrinsic propensity to disassemble – and eventually unfold, initiating a new cycle. Pathological situation in red: Perturbed cellular αS homeostasis, modeled via engineered αS variants, increases (i) the levels of aggregation-prone unfolded monomers in the cytosol (top left; TsixE, E/P mutants) or (ii) the level of membrane-associated monomeric αS (bottom; 3K, KLK, EIV, EGW mutants). While membrane-associated αS accumulation is generally toxic in *in vivo* models, the toxicity of cytosol-accumulated unfolded αS appears to be context specific, e.g., non-toxic in yeast (Volles and Lansbury, 2007), but more toxic than wt αS when virally expressed in mouse SN (Burré et al., 2015).

In combination, the findings by Dettmer et al. (2015a; 2015b), Burré et al. (2015) and Wang et al. (2014) are consistent with native αS multimerization being the result of an intact αS dynamic equilibrium. In such a dynamic equilibrium, αS is expected to efficiently shuttle between cytosol and membranes, i.e., it constantly binds to and gets released from lipid bilayers. Transient interactions of αS monomers with membranes may drive multimerization (Dettmer et al., 2016, 2017), something that recently was observed in an *in vitro* helical αS (tetramer) reconstitution system (Rovere et al., 2018). A possible scenario is that the induced amphipathic αS helices at lipid bilayers may over time cooperatively interact with each other in such a way that the hydrophobic portions of four monomers face each other, resulting in tetramer/multimer formation and simultaneous release from membranes. Too much or too little membrane association could lead to an accumulation of monomers either in the cytosol (e.g., T44P/A89P) or at membranes (e.g., 3K, KLK), respectively (**Figure 3**).

These considerations could help explain how both membrane-enriched αS E46K (Choi et al., 2004) and cytosol-enriched αS A30P (Jensen et al., 1998; Jo et al., 2002) or G51D (Fares et al., 2014) can cause αS dyshomeostasis culminating in the pathogenesis of PD. Indeed, a loss of αS tetramerization/multimer formation had been proposed as a unifying principle between fPD-linked αS mutants: A30P, E46K, H50Q, G51D, and A53T all shifted the dynamic equilibrium away from tetramers/multimers when studied via intact-cell crosslinking and YFP complementation (Dettmer et al., 2015a). This is consistent with the idea that αS multimers may serve as a “safe” (i.e., not aggregation-prone) storage form in the cytoplasm (Gurry et al., 2013; Westphal and Chandra, 2013). The short-term consequences of αS monomer accumulation at membranes (vesicle-rich αS inclusions; pronounced immediate toxicity) or in the cytosol (β-sheet-rich αS aggregates; more subtle/context-dependent toxicity) may differ. One important unsolved question (see above) remains the aggregation state of αS in the “vesicle-rich” inclusions. By EM [wt αS in yeast (Soper et al., 2008) or 3K/KLK αS in neural cells (Dettmer et al., 2017)], such inclusions are not characterized by fibrillar proteinaceous aggregates. In fact, while immunogold analysis clearly shows strong αS enrichment within the vesicle clusters, it is unclear if there is any direct αS-αS interaction at all, be it native or non-native. Assessed via crosslinking and YFP complementation (Dettmer et al., 2015b), αS 3K, KLK, and EIV appeared largely monomeric (at least the crosslinking assays should be able to detect both β-sheet and helical assemblies of αS). However, the aggregation of αS at membranes has been demonstrated (Galvagnion et al., 2015) and only the kinetics of aggregation and toxicity may differ between αS cytosol and membrane accumulation. Eventually, cytosol and membrane pathways of αS dyshomeostasis may converge in common pathological mechanisms, e.g., after the (relatively slow) formation of β-sheet-rich αS aggregates at membranes (Galvagnion et al., 2015). Alternatively, the principle of selective vulnerability (discussed in Walsh and Selkoe, 2016) may help explain why certain brain regions are susceptible to any αS-related insult, while other regions are not. Engineered αS variants, which have more pronounced effects than the fPD-linked mutations, are expected to continue contributing to a better understanding of these phenomena, especially when tested in animal models.

The observation that both increased and decreased membrane binding reduces the αS multimer:monomer ratio may indicate that the folding landscape of wt αS is well balanced to minimize both aggregation-prone unfolded, cytoplasmic monomers and membrane-toxicity causing membrane-associated monomers. Consequently, it may not be possible to further “improve” the *native* wt αS membrane:cytosol and multimer:monomer ratios by protein engineering. *Disturbed* αS homeostasis, however, may be corrected in the future by interfering with αS membrane-interactions. Interestingly, the drug squalamine has recently been reported to displace αS from lipid vesicles, thereby inhibiting aggregation and reducing toxicity (Perni et al., 2017; Pineda and Burré, 2017). However, an A30P carrier may not benefit from such a treatment because A30P already exhibits decreased membrane binding. Also for sporadic PD cases (wt αS), “precision medicine” might be required to determine if the underlying αS dyshomeostasis can be resolved by an increase or a decrease of αS membrane interaction. Alternatively, compounds that can directly stabilize αS tetramers/multimers, analogous to tafamidis in stabilizing transthyretin tetramers in that amyloidosis (Johnson et al., 2012), could arise as “one-size fit all” drugs. The design of such compounds would be facilitated by resolving the structure of tetrameric αS, which appears highly challenging in light of the reported lysis sensitivity of αS multimers (Dettmer et al., 2013; Luth et al., 2015). It is tempting to speculate that membrane-enriched αS variants such as KLK and EIV might even help stabilize αS multimers *in vitro* if we found methods and conditions to release membrane-enriched αS variants from membranes while the hydrophobic faces of their amphipathic helices engage in ordered synuclein-synuclein interactions at the same time. This approach, of course, would only be valid if the mechanism that drives wt αS multimerization *in vivo* is sufficiently similar to this scenario, for which there is some evidence (Wang et al., 2011; Gurry et al., 2013; Rovere et al., 2018).

## αS Function

Several studies have linked αS function to synaptic vesicle trafficking (Abeliovich et al., 2000; Cooper et al., 2006; Larsen et al., 2006; Nemani et al., 2010; Vargas et al., 2014, 2017; Logan et al., 2017). While the exact details are still under debate, this function seems to be tied to transient αS-membrane interactions, which also seem to play a role in αS multimerization (Rovere et al., 2018). Initial reports focused on effects of αS on directly stabilizing curved membrane structures (Kamp et al., 2010; Varkey et al., 2010), similar to BAR domain proteins (DeWitt and Rhoades, 2013; Westphal and Chandra, 2013). More recent studies described direct effects of αS on membrane clustering Wang et al., 2014) or indirect effects of αS on vesicle fusion via the stabilization of vesicle SNARE complexes (Burré et al., 2010, Burré et al., 2014; Almandoz-Gil et al., 2018). Interestingly, both direct and indirect effects have been proposed to be mediated by αS multimerization. Based on biochemical assays, Burré et al. (2014) proposed a dynamic equilibrium between a natively unfolded form in the cytosol and a physiologically functional, multimeric form at membranes, while the latter but not the former acts as a SNARE complex chaperone at the presynaptic terminal. The study by Wang et al. (2014) coupled YFP complementation and confocal microscopy in cultured primary neurons to propose that αS multimerization occurs on synaptic vesicles and is associated with clustering of the vesicles. Wang et al. (2014) employed the αS TsixK variant (**Figure 2H**, left panel) as a negative control and concluded that an αS variant that does not form multimers also does not have vesicle-clustering activity. YFP signals from wt αS-mediated YFP complementation did not only occur at vesicles, but also remained associated with vesicles, leading the authors to postulate that physiological αS multimers have vesicle-clustering activity. If this vesicle-clustering activity is indeed the function of αS, then the excess vesicle-clustering in αS-expressing yeast (Cooper et al., 2006; Soper et al., 2008) may be only quantitatively, but not qualitatively different from the normal αS activity. However, the membrane-enriched αS 3K, KLK, EIV, EGW were largely monomeric when expressed in neuroblastoma cells, as assessed via crosslinking and YFP complementation (Dettmer et al., 2015b), and still exhibited strong vesicle-clustering activity, indicating that pronounced membrane localization of αS may be sufficient to drive vesicle clustering. Thus, 3K, KLK and similar membrane-favoring αS variants may have to be considered constitutively active gain-of-function mutants with regard to vesicle-clustering activity. Cytosol-enriched αS mutations such as A11P/V70 and T44P/A89P as well as the folding-incompetent TsixK, on the other hand, would be loss-of function mutations (**Figure 2**). In another study, the generation of membrane curvature (another activity of αS that is potentially related to its vesicle-clustering activity), has been assigned to monomeric αS at membranes (Westphal and Chandra, 2013). Conversely, soluble tetrameric αS was described in the same study as a passive storage form of the protein (Westphal and Chandra, 2013). The in part contradictory results summarized here might be resolved eventually by a model in which the protein’s ability to rapidly switch between different localizations and folding/assembly states mediates its activity. In light of such a model, the question if monomeric or multimeric αS represents the functional form of the protein becomes obsolete. An analogy for such a scenario would be SNARE proteins that switch between monomeric and (hetero)tetrameric states, thereby driving vesicle fusion events. In the meantime, the αS variants reviewed here represent valuable tools for the field’s attempt to better understand αS functions and assign certain aspects of those functions to certain conformations of the protein. As an example, it would be interesting to test if the exocytic fusion-pore dilating effect of wt αS (Logan et al., 2017) is increased or decreased for membrane-enriched αS variants and if cytosol-enriched variants have the opposite effect. In addition to the membrane-associated functions of αS, engineered soluble αS mutants offer opportunities for testing and identifying cytosolic functions of the protein.

## An Attempt to Categorize αS Mutations

A simplified wheel diagram of a membrane-induced amphipathic helix of αS (**Figure 1**) helps categorize αS variants into two classes: membrane-enriched and cytosol-enriched variants. Membrane-enriched αS variants stabilize the formation of the αS membrane-induced amphipathic helix, cytosol-enriched variants destabilize the helix.

**FIGURE 4:**
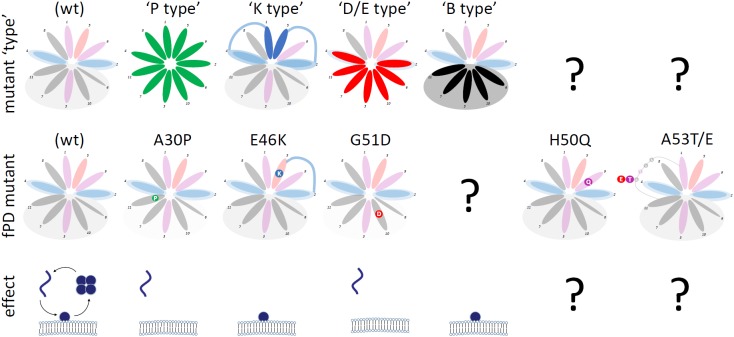
Categories of αS Mutants. Wt αS (shaded; over-simplified by choosing the color-code of the most prevalent aa for each 11-aa repeat position) and αS variants (theoretical: top row; fPD: middle row) are shown. “Mutant type” (upper row) highlights the positions at which the respective mutations are expected to exert the proposed effects. “Effect” (bottom row) shows the (exaggerated) effect of the mutations on αS homeostasis: monomers accumulate at the membrane or in the cytosol.

Cytosol-enriched αS variants can be further classified into (**Figure 4**):

- “P-type” variants: substitutions with proline (P) residues prevent helix formation, leading to the accumulation of cytosolic αS, which is likely unfolded, monomeric and aggregation-prone. P, known as a “helix-breaker”, should have this effect at any position in αS. fPD-linked αS A30P (Krüger et al., 1998; Jo et al., 2002) falls into this category; see **Figures 2C,E** for engineered analogs/amplifications.
- “D/E-type” variants: substitutions with aspartate (D) or glutamate (E) residues cause repulsion from lipid acyl chains (D/E in the hydrophobic half of the αS helix). As a result, cytosolic αS accumulates, which is likely unfolded, monomeric and aggregation-prone. fPD-linked αS G51D (Kiely et al., 2013; Lesage et al., 2013; Fares et al., 2014) is one example; see **Figures 2C,H** for engineered analogs/amplifications. D/E substitutions should have similar effects in the “lysine wings” (**Figure 2D**).

The effect of “P-” and “D/E-type” variants on αS toxicity appears to be model- and context-specific and ranges from less toxic than wt in yeast (Volles and Lansbury, 2007) to more toxic than wt in virally injected mouse SN (Burré et al., 2015). This discrepancy, which may have to do with differences in β-sheet-rich aggregation in different cellular environments, will require further elucidation. Of course, human genetics underlines the long-term toxicity of both types: the fPD-linked variants A30P (Krüger et al., 1998; Jo et al., 2002) and G51D (Kiely et al., 2013; Lesage et al., 2013; Fares et al., 2014) cause PD. However, A30P and G51D are likely not readily toxic in yeast or (simple) cellular models, consistent with membrane interactions being important for yeast αS toxicity (Volles and Lansbury, 2007).

Membrane-enriched αS variants can be further classified into (**Figure 4**):

- “K-type variants”: substitutions with lysine (K) residues at position 1 or 5 of each 11-aa repeat presumably lead to an energetically favorable interaction with lipid headgroups, in analogy to what has been proposed for fPD-linked αS E46K (Perlmutter et al., 2009) (**Figure 2B**). The result of a stabilized αS membrane-induced amphipathic helix is the accumulation of membrane-associated, helically folded (and monomeric) αS. See **Figure 2F** for analogs and exaggerations of E46K. It should be noted that “K-type” mutations are highly position-specific. All variants discussed here are located at positions 1 and 5 of each 11-aa repeat. A similar effect may occur at positions 8 and 9, but this remains to be tested. The positively charged histidine (position 9 in repeat 4, aa 50 in αS) may stabilize the αS helix in the environment of negatively charged lipid headgroups and therefore the fPD-linked H50Q (Appel-Cresswell et al., 2013) (**Figure 4**, second column from the right) may (slightly) repel αS from membranes. The presence of positively charged K (or R) at the (hydrophobic) positions 3, 6, 7, 10, or 11 will interfere with helix formation and lead to repulsion or a different mode of membrane binding [as discussed by Perrin et al. (2000); **Figure 2H**, left panel].
- “B-type variants”: substitutions with “bulky” residues (L, I, W, F) in the hydrophobic half of the αS amphipathic helix enhance hydrophobic interactions, leading to increased membrane dwell-time (**Figure 2G**). “B-type” αS variants do not have a counterpart in an fPD-linked variant. This could be coincidence or indicative of a strong toxicity that has prevented the presence of such variants in humans.

“K-type” αS variants show consistent toxicity in yeast (Volles and Lansbury, 2007), cellular (Dettmer et al., 2015a), and animal (Winner et al., 2011) models, which should most likely also apply to “B-type” variants (Dettmer et al., 2015b). The pronounced toxicity of these variants, however, is possibly caused by disrupting membrane integrity and vesicle trafficking (Dettmer et al., 2017), and the relationship to β-sheet-rich aggregation will require further elucidation. Thus far, it is not clear if there is any direct αS-αS interaction at all in the vesicle-rich αS inclusions. Recent work (Fusco et al., 2016) suggests that excess αS monomers, that each are in contact with two vesicle membranes, could directly cause aberrant vesicle clustering. Importantly, however, the fPD-linked E46K, a “K-type” mutant, produces typical proteinaceous inclusions, Lewy bodies, in patient brains (Zarranz et al., 2004), arguing against an E46K-specific pathogenic mechanism that is entirely independent of αS proteinaceous aggregation.

## Limitations of the Presented Studies and Their Interpretation Based on a Simplified αS Membrane-Induced Helix Model

The studies presented and discussed here typically rely on the overexpression of αS, sometimes fused to whole or split fluorescent proteins, in human/rodent cells or even in yeast, an organism that does not possess synuclein. Excess/ectopic expression and the modification with tags that are larger than αS itself may of course affect the localization and structure of the studied variants. Nonetheless, all presented αS variants were compared to wt αS expressed under the same conditions and, therefore, the conclusions relative to wt αS can be expected to be meaningful. Moreover, many key observations such as the toxic effect of adding positive charges to positions 1 or 5 of the 11-aa repeat have been confirmed independent of tagging (untagged, YFP-tagged) or model system (yeast, transfected neural cells, stereotactic viral expression in mouse substantia nigra). However, the effect of expressing folding-impaired P-mutant αS ranges from less toxic (yeast) to more toxic (stereotactic viral expression in mouse substantia nigra) and, while it is obvious to consider the findings in the rodent *in vivo* system more relevant, the study of the “A30P-like” αS toxicity remains challenging.

It also has to be noted again that the synoptic considerations in this review are based on a *simplified* wheel model of αS membrane-induced amphipathic helix formation. The model ignores the “ATVA” interrupting sequence, which leads to a shift (repeats 5–7 relative to repeats 1–4) that is not reflected. The fPD-linked mutations A53T (Polymeropoulos et al., 1997) (ATVA becomes TTVA) and A53E (Pasanen et al., 2014) (ATVA becomes ETVA) cannot be assessed via the simplified wheel model (**Figure 4**, right column). At least for A53E, reduced membrane binding has been reported (Ghosh et al., 2014), while the membrane binding of A53T may be similar to wt (Bussell and Eliezer, 2004). Positions 8 and 9 in the 11-aa repeats seem to be understudied. While located in the hydrophilic half of the αS helix, the nature of the aa found at position 8 are surprisingly hydrophobic, especially in repeats 1–4 (V, A, L, V). This may have to do with membrane-interaction-independent requirements for helix formation and may be addressed in future studies. At position 9, aa with very different characteristics are found: A, E, Y, H, N, A, S. The only positively charged aa, H, is mutated in fPD-linked H50Q and assessing this effect based on the simplified helix model is not straightforward (see above). Moreover, the simplified wheel diagrams in **Figure 2** only depict aa 9–89 while regions up to aa 97 (Fusco et al., 2016) and also the very N-terminus (Bartels et al., 2010) have been proposed to be involved in membrane binding.

It cannot be ruled out that some of the mutants discussed here have very specific effects unrelated to their position in the membrane-induced αS amphipathic helix. For example, E35K and E57K (**Figure 2F**) have been reported to have specific effects on αS aggregation (oligomer formation instead of fast fibrillization) *in vitro*, in the absence of membranes. However, based on the existing data, it appears reasonable to predict that E13K, E83K and, possibly even E105K (all at position 5 of the 11-aa repeat) have similar effects as E46K. The same should apply to E20K (position 1 of 11-aa repeat #2) and mutants generated by replacing the non-E amino acids at positions 1 and 5 of the 11-aa repeats, such as S9K or Q24K, will likely also behave similarly.

The predictive value of the simplified αS wheel diagram will continue to be tested by new strategic αS mutants that researchers will create and possibly even by newly found fPD-linked αS variants. fPD-linked A53T and A53E (see above), which are not in the 11-aa repeat may very well have unique effects and the effect of H50Q, where the only H in αS is mutated, is hard to assess based on the simplified model (see above). H50Q (Khalaf et al., 2014; Rutherford et al., 2014) and A53T (Conway et al., 1998) were reported to form amyloid more readily *in vitro* and – leaving the membrane-induced helix model aside – increased aggregation-propensity of soluble αS in an otherwise “normal” equilibrium of membrane-bound and soluble αS represents a sufficient explanation for long-term toxicity. It should, again, be noted though that the fPD variants A30P, E46K, H50Q, G51D, and A53T were reported to have an αS multimer:monomer ratio that was shifted toward more monomer, suggesting that a reduced multimer:monomer ratio could be the “unifying principle” of αS dyshomeostasis, independent of whether excess monomers accumulate at membranes or in the cytosol (Dettmer et al., 2015a). Lastly, important aspects of αS biology were beyond the scope of this review, which focused mainly on strategic aa mutations in the αS 11-aa repeats studied *in cellulo*. The composition of (vesicular) membranes has been repeatedly shown to influence αS binding (e.g., Nuscher et al., 2004), and the effect of certain αS fPD mutations on, e.g., membrane binding may depend on the exact nature of target membranes. Similarly, certain post-translational modifications such as S129 phosphorylation have been proposed to be an important aspect of αS homeostasis and they have been studied, e.g., via aa substitutions that prevent the respective modifications (e.g., Lázaro et al., 2014). Such aa substitutions were not discussed here. αS function was not the main focus of this review because only two of the key studies presented explicitly addressed it; the proposed function were vesicle clustering (Wang et al., 2014) and SNARE assembly at vesicles (Burré et al., 2012). However, future studies related to other proposed cellular αS interactions such as that with mitochondria (e.g., Kamp et al., 2010) may also benefit from analyzing strategic αS variants.

## Author Contributions

UD wrote the review.

## Conflict of Interest Statement

The author declares that the research was conducted in the absence of any commercial or financial relationships that could be construed as a potential conflict of interest.

## References

[B1] Abeliovich A. Schmitz Y. Fariñas I. Choi-Lundberg D. Ho W. H. Castillo P. E. (2000). Mice lacking alpha-synuclein display functional deficits in the nigrostriatal dopamine system. *Neuron* 25 239–252. 10.1016/S0896-6273(00)80886-7 10707987

[B2] Alderson T. R. Bax A. (2016). Parkinson’s disease: disorder in the court. *Nature* 530 38–39. 10.1038/nature16871 26808901

[B3] Almandoz-Gil L. Persson E. Lindström V. Ingelsson M. Erlandsson A. Bergström J. (2018). In situ proximity ligation assay reveals co-localization of alpha-synuclein and snare proteins in murine primary neurons. *Front. Neurol.* 9:180. 10.3389/fneur.2018.00180 29623065 PMC5874290

[B4] Appel-Cresswell S. Vilarino-Guell C. Encarnacion M. Sherman H. Yu I. Shah B. (2013). Alpha-synuclein p.H50Q, a novel pathogenic mutation for Parkinson’s disease. *Mov. Disord. Off. J. Mov. Disord. Soc.* 28 811–813. 10.1002/mds.25421 23457019

[B5] Bartels T. Ahlstrom L. S. Leftin A. Kamp F. Haass C. Brown M. F. (2010). The N-terminus of the intrinsically disordered protein α-synuclein triggers membrane binding and helix folding. *Biophys. J.* 99 2116–2124. 10.1016/j.bpj.2010.06.035 20923645 PMC3042581

[B6] Bartels T. Choi J. G. Selkoe D. J. (2011). α-Synuclein occurs physiologically as a helically folded tetramer that resists aggregation. *Nature* 477 107–110. 10.1038/nature10324 21841800 PMC3166366

[B7] Bendor J. T. Logan T. P. Edwards R. H. (2013). The function of α-synuclein. *Neuron* 79 1044–1066. 10.1016/j.neuron.2013.09.004 24050397 PMC3866954

[B8] Bertoncini C. W. Jung Y.-S. Fernandez C. O. Hoyer W. Griesinger C. Jovin T. M. (2005). Release of long-range tertiary interactions potentiates aggregation of natively unstructured alpha-synuclein. *Proc. Natl. Acad. Sci. U.S.A.* 102 1430–1435. 10.1073/pnas.0407146102 15671169 PMC547830

[B9] Binolfi A. Theillet F.-X. Selenko P. (2012). Bacterial in-cell NMR of human α-synuclein: a disordered monomer by nature? *Biochem. Soc. Trans.* 40 950–954. 22988846 10.1042/BST20120096

[B10] Burré J. Sharma M. Südhof T. C. (2012). Systematic mutagenesis of α-synuclein reveals distinct sequence requirements for physiological and pathological activities. *J. Neurosci. Off. J. Soc. Neurosci.* 32 15227–15242. 10.1523/JNEUROSCI.3545-12.2012 PMC3506191 23100443

[B11] Burré J. Sharma M. Südhof T. C. (2014). α-Synuclein assembles into higher-order multimers upon membrane binding to promote SNARE complex formation. *Proc. Natl. Acad. Sci. U.S.A.* 111 E4274–E4283. 10.1073/pnas.1416598111 25246573 PMC4210039

[B12] Burré J. Sharma M. Südhof T. C. (2015). Definition of a Molecular Pathway Mediating α-Synuclein Neurotoxicity. *J. Neurosci. Off. J. Soc. Neurosci.* 35 5221–5232. 10.1523/JNEUROSCI.4650-14.2015 PMC4380997 25834048

[B13] Burré J. Sharma M. Tsetsenis T. Buchman V. Etherton M. R. Südhof T. C. (2010). Alpha-synuclein promotes SNARE-complex assembly in vivo and in vitro. *Science* 329 1663–1667. 10.1126/science.1195227 20798282 PMC3235365

[B14] Bussell R. Eliezer D. (2004). Effects of Parkinson’s disease-linked mutations on the structure of lipid-associated alpha-synuclein. *Biochemistry* 43 4810–4818. 10.1021/bi036135+ 15096050

[B15] Bussell R. Ramlall T. F. Eliezer D. (2005). Helix periodicity, topology, and dynamics of membrane-associated alpha-synuclein. *Protein Sci. Publ. Protein Soc.* 14 862–872. 10.1110/ps.041255905 15741347 PMC2253433

[B16] Chen R. H. C. Wislet-Gendebien S. Samuel F. Visanji N. P. Zhang G. Marsilio D. (2013). α-Synuclein membrane association is regulated by the Rab3a recycling machinery and presynaptic activity. *J. Biol. Chem.* 288 7438–7449. 10.1074/jbc.M112.439497 23344955 PMC3597785

[B17] Choi W. Zibaee S. Jakes R. Serpell L. C. Davletov B. Crowther R. A. (2004). Mutation E46K increases phospholipid binding and assembly into filaments of human alpha-synuclein. *FEBS Lett.* 576 363–368. 10.1016/j.febslet.2004.09.038 15498564

[B18] Chung C. Y. Khurana V. Auluck P. K. Tardiff D. F. Mazzulli J. R. Soldner F. (2013). Identification and rescue of α-synuclein toxicity in Parkinson patient-derived neurons. *Science* 342 983–987. 10.1126/science.1245296 24158904 PMC4022187

[B19] Conway K. A. Harper J. D. Lansbury P. T. (1998). Accelerated in vitro fibril formation by a mutant alpha-synuclein linked to early-onset Parkinson disease. *Nat. Med.* 4 1318–1320. 10.1038/3311 9809558

[B20] Conway K. A. Harper J. D. Lansbury P. T. (2000). Fibrils formed in vitro from alpha-synuclein and two mutant forms linked to Parkinson’s disease are typical amyloid. *Biochemistry* 39 2552–2563. 10.1021/bi991447r 10704204

[B21] Cooper A. A. Gitler A. D. Cashikar A. Haynes C. M. Hill K. J. Bhullar B. (2006). Alpha-synuclein blocks ER-Golgi traffic and Rab1 rescues neuron loss in Parkinson’s models. *Science* 313 324–328. 10.1126/science.1129462 16794039 PMC1983366

[B22] Davidson W. S. Jonas A. Clayton D. F. George J. M. (1998). Stabilization of alpha-synuclein secondary structure upon binding to synthetic membranes. *J. Biol. Chem.* 273 9443–9449. 10.1074/jbc.273.16.9443 9545270

[B23] Dettmer U. Newman A. J. Luth E. S. Bartels T. Selkoe D. (2013). In vivo cross-linking reveals principally oligomeric forms of α-synuclein and β-synuclein in neurons and non-neural cells. *J. Biol. Chem.* 288 6371–6385. 10.1074/jbc.M112.403311 23319586 PMC3585072

[B24] Dettmer U. Newman A. J. Soldner F. Luth E. S. Kim N. C. von Saucken V. E. (2015a). Parkinson-causing α-synuclein missense mutations shift native tetramers to monomers as a mechanism for disease initiation. *Nat. Commun.* 6:7314. 10.1038/ncomms8314 26076669 PMC4490410

[B25] Dettmer U. Newman A. J. von Saucken V. E. Bartels T. Selkoe D. (2015b). KTKEGV repeat motifs are key mediators of normal α-synuclein tetramerization: their mutation causes excess monomers and neurotoxicity. *Proc. Natl. Acad. Sci. U.S.A.* 112 9596–9601. 10.1073/pnas.1505953112 26153422 PMC4534262

[B26] Dettmer U. Ramalingam N. von Saucken V. E. Kim T.-E. Newman A. J. Terry-Kantor E. (2017). Loss of native α-synuclein multimerization by strategically mutating its amphipathic helix causes abnormal vesicle interactions in neuronal cells. *Hum. Mol. Genet.* 26 3466–3481. 10.1093/hmg/ddx227 28911198 PMC5884392

[B27] Dettmer U. Selkoe D. Bartels T. (2016). New insights into cellular α-synuclein homeostasis in health and disease. *Curr. Opin. Neurobiol.* 36 15–22. 10.1016/j.conb.2015.07.007 26282834

[B28] DeWitt D. C. Rhoades E. (2013). α-Synuclein can inhibit SNARE-mediated vesicle fusion through direct interactions with lipid bilayers. *Biochemistry* 52 2385–2387. 10.1021/bi4002369 23528131 PMC3640401

[B29] Eliezer D. Kutluay E. Bussell R. Browne G. (2001). Conformational properties of alpha-synuclein in its free and lipid-associated states. *J. Mol. Biol.* 307 1061–1073. 10.1006/jmbi.2001.4538 11286556

[B30] Fares M.-B. Ait-Bouziad N. Dikiy I. Mbefo M. K. Jovièiæ A. Kiely A. (2014). The novel Parkinson’s disease linked mutation G51D attenuates in vitro aggregation and membrane binding of α-synuclein, and enhances its secretion and nuclear localization in cells. *Hum. Mol. Genet.* 23 4491–4509. 10.1093/hmg/ddu165 24728187 PMC4119404

[B31] Fauvet B. Mbefo M. K. Fares M.-B. Desobry C. Michael S. Ardah M. T. (2012). α-Synuclein in central nervous system and from erythrocytes, mammalian cells, and *Escherichia coli* exists predominantly as disordered monomer. *J. Biol. Chem.* 287 15345–15364. 10.1074/jbc.M111.318949 22315227 PMC3346117

[B32] Fortin D. L. Nemani V. M. Nakamura K. Edwards R. H. (2010). The behavior of alpha-synuclein in neurons. *Mov. Disord. Off. J. Mov. Disord. Soc.* 25(Suppl. 1), S21–S26. 10.1002/mds.22722 20187244

[B33] Fuchs J. Tichopad A. Golub Y. Munz M. Schweitzer K. J. Wolf B. (2008). Genetic variability in the SNCA gene influences alpha-synuclein levels in the blood and brain. *FASEB J. Off. Publ. Fed. Am. Soc. Exp. Biol.* 22 1327–1334. 18162487 10.1096/fj.07-9348com

[B34] Fusco G. Pape T. Stephens A. D. Mahou P. Costa A. R. Kaminski C. F. (2016). Structural basis of synaptic vesicle assembly promoted by α-synuclein. *Nat. Commun.* 7:12563. 10.1038/ncomms12563 27640673 PMC5031799

[B35] Galvagnion C. Buell A. K. Meisl G. Michaels T. C. T. Vendruscolo M. Knowles T. P. J. (2015). Lipid vesicles trigger α-synuclein aggregation by stimulating primary nucleation. *Nat. Chem. Biol.* 11 229–234. 10.1038/nchembio.1750 25643172 PMC5019199

[B36] George J. M. Jin H. Woods W. S. Clayton D. F. (1995). Characterization of a novel protein regulated during the critical period for song learning in the zebra finch. *Neuron* 15 361–372. 10.1016/0896-6273(95)90040-3 7646890

[B37] Ghosh D. Sahay S. Ranjan P. Salot S. Mohite G. M. Singh P. K. (2014). The newly discovered Parkinson’s disease associated Finnish mutation (A53E) attenuates α-synuclein aggregation and membrane binding. *Biochemistry* 53 6419–6421. 10.1021/bi5010365 25268550

[B38] Gould N. Mor D. E. Lightfoot R. Malkus K. Giasson B. Ischiropoulos H. (2014). Evidence of native α-synuclein conformers in the human brain. *J. Biol. Chem.* 289 7929–7934. 10.1074/jbc.C113.538249 24474688 PMC3953303

[B39] Gurry T. Ullman O. Fisher C. K. Perovic I. Pochapsky T. Stultz C. M. (2013). The dynamic structure of α-synuclein multimers. *J. Am. Chem. Soc.* 135 3865–3872. 10.1021/ja310518p 23398399

[B40] Iljina M. Tosatto L. Choi M. L. Sang J. C. Ye Y. Hughes C. D. (2016). Arachidonic acid mediates the formation of abundant alpha-helical multimers of alpha-synuclein. *Sci. Rep.* 6:33928. 10.1038/srep33928 27671749 PMC5037366

[B41] Íñigo-Marco I. Valencia M. Larrea L. Bugallo R. Martínez-Goikoetxea M. Zuriguel I. (2017). E46K α-synuclein pathological mutation causes cell-autonomous toxicity without altering protein turnover or aggregation. *Proc. Natl. Acad. Sci. U.S.A.* 114 E8274–E8283. 10.1073/pnas.1703420114 28900007 PMC5625897

[B42] Jao C. C. Der-Sarkissian A. Chen J. Langen R. (2004). Structure of membrane-bound alpha-synuclein studied by site-directed spin labeling. *Proc. Natl. Acad. Sci. U.S.A.* 101 8331–8336. 10.1073/pnas.0400553101 15155902 PMC420394

[B43] Jao C. C. Hegde B. G. Chen J. Haworth I. S. Langen R. (2008). Structure of membrane-bound alpha-synuclein from site-directed spin labeling and computational refinement. *Proc. Natl. Acad. Sci. U.S.A.* 105 19666–19671. 10.1073/pnas.0807826105 19066219 PMC2605001

[B44] Jarosz D. F. Khurana V. (2017). Specification of Physiologic and Disease States by Distinct Proteins and Protein Conformations. *Cell* 171 1001–1014. 10.1016/j.cell.2017.10.047 29149602

[B45] Jensen M. B. Bhatia V. K. Jao C. C. Rasmussen J. E. Pedersen S. L. Jensen K. J. (2011). Membrane curvature sensing by amphipathic helices: a single liposome study using alpha-synuclein and annexin B12. *J. Biol. Chem.* 286 42603–42614. 10.1074/jbc.M111.271130 21953452 PMC3234936

[B46] Jensen P. H. Nielsen M. S. Jakes R. Dotti C. G. Goedert M. (1998). Binding of alpha-synuclein to brain vesicles is abolished by familial Parkinson’s disease mutation. *J. Biol. Chem.* 273 26292–26294. 10.1074/jbc.273.41.26292 9756856

[B47] Jo E. Fuller N. Rand R. P. St George-Hyslop P. Fraser P. E. (2002). Defective membrane interactions of familial Parkinson’s disease mutant A30P alpha-synuclein. *J. Mol. Biol.* 315 799–807. 10.1006/jmbi.2001.5269 11812148

[B48] Johnson S. M. Connelly S. Fearns C. Powers E. T. Kelly J. W. (2012). The transthyretin amyloidoses: from delineating the molecular mechanism of aggregation linked to pathology to a regulatory-agency-approved drug. *J. Mol. Biol.* 421 185–203. 10.1016/j.jmb.2011.12.060 22244854 PMC3350832

[B49] Kahle P. J. Neumann M. Ozmen L. Muller V. Jacobsen H. Schindzielorz A. (2000). Subcellular localization of wild-type and Parkinson’s disease-associated mutant alpha -synuclein in human and transgenic mouse brain. *J. Neurosci. Off. J. Soc. Neurosci.* 20 6365–6373. 10.1523/JNEUROSCI.20-17-06365.2000 PMC6772969 10964942

[B50] Kamp F. Exner N. Lutz A. K. Wender N. Hegermann J. Brunner B. (2010). Inhibition of mitochondrial fusion by α-synuclein is rescued by PINK1, Parkin and DJ-1. *EMBO J.* 29 3571–3589. 10.1038/emboj.2010.223 20842103 PMC2964170

[B51] Kara E. Lewis P. A. Ling H. Proukakis C. Houlden H. Hardy J. (2013). α-Synuclein mutations cluster around a putative protein loop. *Neurosci. Lett.* 546 67–70. 10.1016/j.neulet.2013.04.058 23669636 PMC3694303

[B52] Khalaf O. Fauvet B. Oueslati A. Dikiy I. Mahul-Mellier A.-L. Ruggeri F. S. (2014). The H50Q mutation enhances α-synuclein aggregation, secretion, and toxicity. *J. Biol. Chem.* 289 21856–21876. 10.1074/jbc.M114.553297 24936070 PMC4139205

[B53] Kiely A. P. Asi Y. T. Kara E. Limousin P. Ling H. Lewis P. (2013). α-Synucleinopathy associated with G51D SNCA mutation: a link between Parkinson’s disease and multiple system atrophy? *Acta Neuropathol.* 125 753–769. 10.1007/s00401-013-1096-7 23404372 PMC3681325

[B54] Kim Y. S. Laurine E. Woods W. Lee S.-J. (2006). A novel mechanism of interaction between alpha-synuclein and biological membranes. *J. Mol. Biol.* 360 386–397. 10.1016/j.jmb.2006.05.004 16762368

[B55] Krüger R. Kuhn W. Müller T. Woitalla D. Graeber M. Kösel S. (1998). Ala30Pro mutation in the gene encoding alpha-synuclein in Parkinson’s disease. *Nat. Genet.* 18 106–108. 10.1038/ng0298-106 9462735

[B56] Larsen K. E. Schmitz Y. Troyer M. D. Mosharov E. Dietrich P. Quazi A. Z. (2006). Alpha-synuclein overexpression in PC12 and chromaffin cells impairs catecholamine release by interfering with a late step in exocytosis. *J. Neurosci. Off. J. Soc. Neurosci.* 26 11915–11922. 10.1523/JNEUROSCI.3821-06.2006 17108165 PMC6674868

[B57] Lázaro D. F. Rodrigues E. F. Langohr R. Shahpasandzadeh H. Ribeiro T. Guerreiro P. (2014). Systematic comparison of the effects of alpha-synuclein mutations on its oligomerization and aggregation. *PLoS Genet.* 10:e1004741. 10.1371/journal.pgen.1004741 25393002 PMC4230739

[B58] Lesage S. Anheim M. Letournel F. Bousset L. Honoré A. Rozas N. (2013). G51D α-synuclein mutation causes a novel parkinsonian-pyramidal syndrome. *Ann. Neurol.* 73 459–471. 10.1002/ana.23894 23526723

[B59] Logan T. Bendor J. Toupin C. Thorn K. Edwards R. H. (2017). α-Synuclein promotes dilation of the exocytotic fusion pore. *Nat. Neurosci.* 20 681–689. 10.1038/nn.4529 28288128 PMC5404982

[B60] Londos C. Brasaemle D. L. Schultz C. J. Segrest J. P. Kimmel A. R. (1999). Perilipins, ADRP, and other proteins that associate with intracellular neutral lipid droplets in animal cells. *Semin. Cell Dev. Biol.* 10 51–58. 10.1006/scdb.1998.0275 10355028

[B61] Luth E. S. Bartels T. Dettmer U. Kim N. C. Selkoe D. J. (2015). Purification of α-synuclein from human brain reveals an instability of endogenous multimers as the protein approaches purity. *Biochemistry* 54 279–292. 10.1021/bi501188a 25490121 PMC4303315

[B62] Mbefo M. K. Fares M.-B. Paleologou K. Oueslati A. Yin G. Tenreiro S. (2015). Parkinson disease mutant E46K enhances α-synuclein phosphorylation in mammalian cell lines, in yeast, and in vivo. *J. Biol. Chem.* 290 9412–9427. 10.1074/jbc.M114.610774 25657004 PMC4392248

[B63] Meeus B. Theuns J. Van Broeckhoven C. (2012). The genetics of dementia with Lewy bodies: what are we missing? *Arch. Neurol.* 69 1113–1118. 10.1001/archneurol.2011.3678 22635379

[B64] Middleton E. R. Rhoades E. (2010). Effects of curvature and composition on alpha-synuclein binding to lipid vesicles. *Biophys. J.* 99 2279–2288. 10.1016/j.bpj.2010.07.056 20923663 PMC3042580

[B65] Nemani V. M. Lu W. Berge V. Nakamura K. Onoa B. Lee M. K. (2010). Increased expression of alpha-synuclein reduces neurotransmitter release by inhibiting synaptic vesicle reclustering after endocytosis. *Neuron* 65 66–79. 10.1016/j.neuron.2009.12.023 20152114 PMC3119527

[B66] Nuscher B. Kamp F. Mehnert T. Odoy S. Haass C. Kahle P. J. (2004). Alpha-synuclein has a high affinity for packing defects in a bilayer membrane: a thermodynamics study. *J. Biol. Chem.* 279 21966–21975. 10.1074/jbc.M401076200 15028717

[B67] Pasanen P. Myllykangas L. Siitonen M. Raunio A. Kaakkola S. Lyytinen J. (2014). Novel α-synuclein mutation A53E associated with atypical multiple system atrophy and Parkinson’s disease-type pathology. *Neurobiol. Aging* 35 2180.e1–2180.e5. 10.1016/j.neurobiolaging.2014.03.024 24746362

[B68] Perlmutter J. D. Braun A. R. Sachs J. N. (2009). Curvature dynamics of alpha-synuclein familial Parkinson disease mutants: molecular simulations of the micelle- and bilayer-bound forms. *J. Biol. Chem.* 284 7177–7189. 10.1074/jbc.M808895200 19126542 PMC2652287

[B69] Perni M. Galvagnion C. Maltsev A. Meisl G. Müller M. B. D. Challa P. K. (2017). A natural product inhibits the initiation of α-synuclein aggregation and suppresses its toxicity. *Proc. Natl. Acad. Sci. U.S.A.* 114 E1009–E1017. 10.1073/pnas.1610586114 28096355 PMC5307473

[B70] Perrin R. J. Woods W. S. Clayton D. F. George J. M. (2000). Interaction of human alpha-Synuclein and Parkinson’s disease variants with phospholipids. Structural analysis using site-directed mutagenesis. *J. Biol. Chem.* 275 34393–34398. 10.1074/jbc.M004851200 10952980

[B71] Pineda A. Burré J. (2017). Modulating membrane binding of α-synuclein as a therapeutic strategy. *Proc. Natl. Acad. Sci. U.S.A.* 114 1223–1225. 10.1073/pnas.1620159114 28126719 PMC5307480

[B72] Polymeropoulos M. H. Lavedan C. Leroy E. Ide S. E. Dehejia A. Dutra A. (1997). Mutation in the alpha-synuclein gene identified in families with Parkinson’s disease. *Science* 276 2045–2047. 10.1126/science.276.5321.2045 9197268

[B73] Pranke I. M. Morello V. Bigay J. Gibson K. Verbavatz J.-M. Antonny B. (2011). α-Synuclein and ALPS motifs are membrane curvature sensors whose contrasting chemistry mediates selective vesicle binding. *J. Cell Biol.* 194 89–103. 10.1083/jcb.201011118 21746853 PMC3135411

[B74] Proukakis C. Dudzik C. G. Brier T. MacKay D. S. Cooper J. M. Millhauser G. L. (2013). A novel α-synuclein missense mutation in Parkinson disease. *Neurology* 80 1062–1064. 10.1212/WNL.0b013e31828727ba 23427326 PMC3653201

[B75] Rovere M. Sanderson J. B. Fonseca-Ornelas L. Patel D. S. Bartels T. (2018). Refolding of helical soluble α-synuclein through transient interaction with lipid interfaces. *FEBS Lett.* 592 1464–1472. 10.1002/1873-3468.13047 29633780

[B76] Rutherford N. J. Moore B. D. Golde T. E. Giasson B. I. (2014). Divergent effects of the H50Q and G51D SNCA mutations on the aggregation of α-synuclein. *J. Neurochem.* 131 859–867. 10.1111/jnc.12806 24984882

[B77] Scott D. Roy S. (2012). α-Synuclein inhibits intersynaptic vesicle mobility and maintains recycling-pool homeostasis. *J. Neurosci. Off. J. Soc. Neurosci.* 32 10129–10135. 10.1523/JNEUROSCI.0535-12.2012 PMC3426499 22836248

[B78] Singleton A. B. Farrer M. Johnson J. Singleton A. Hague S. Kachergus J. (2003). alpha-Synuclein locus triplication causes Parkinson’s disease. *Science* 302:841. 10.1126/science.1090278 14593171

[B79] Sipe J. D. Benson M. D. Buxbaum J. N. Ikeda S. Merlini G. Saraiva M. J. M. (2014). Nomenclature 2014: amyloid fibril proteins and clinical classification of the amyloidosis. *Amyloid* 21 221–224. 10.3109/13506129.2014.964858 25263598

[B80] Soper J. H. Roy S. Stieber A. Lee E. Wilson R. B. Trojanowski J. Q. (2008). Alpha-synuclein-induced aggregation of cytoplasmic vesicles in *Saccharomyces cerevisiae*. *Mol. Biol. Cell* 19 1093–1103. 10.1091/mbc.E07-08-0827 18172022 PMC2262993

[B81] Spillantini M. G. Schmidt M. L. Lee V. M. Trojanowski J. Q. Jakes R. Goedert M. (1997). Alpha-synuclein in Lewy bodies. *Nature* 388 839–840. 10.1038/42166 9278044

[B82] Tanner C. M. Goldman S. M. (1996). Epidemiology of Parkinson’s disease. *Neurol. Clin.* 14 317–335. 10.1016/S0733-8619(05)70259-0 8827174 PMC7173037

[B83] Theillet F.-X. Binolfi A. Bekei B. Martorana A. Rose H. M. Stuiver M. (2016). Structural disorder of monomeric α-synuclein persists in mammalian cells. *Nature* 530 45–50. 10.1038/nature16531 26808899

[B84] Ulmer T. S. Bax A. (2005). Comparison of structure and dynamics of micelle-bound human alpha-synuclein and Parkinson disease variants. *J. Biol. Chem.* 280 43179–43187. 10.1074/jbc.M507624200 16166095

[B85] Vargas K. J. Makani S. Davis T. Westphal C. H. Castillo P. E. Chandra S. S. (2014). Synucleins regulate the kinetics of synaptic vesicle endocytosis. *J. Neurosci. Off. J. Soc. Neurosci.* 34 9364–9376. 10.1523/JNEUROSCI.4787-13.2014 25009269 PMC4087213

[B86] Vargas K. J. Schrod N. Davis T. Fernandez-Busnadiego R. Taguchi Y. V. Laugks U. (2017). Synucleins have multiple effects on presynaptic architecture. *Cell Rep.* 18 161–173. 10.1016/j.celrep.2016.12.023 28052246 PMC5510332

[B87] Varkey J. Isas J. M. Mizuno N. Jensen M. B. Bhatia V. K. Jao C. C. (2010). Membrane curvature induction and tubulation are common features of synucleins and apolipoproteins. *J. Biol. Chem.* 285 32486–32493. 10.1074/jbc.M110.139576 20693280 PMC2952250

[B88] Volles M. J. Lansbury P. T. (2007). Relationships between the sequence of alpha-synuclein and its membrane affinity, fibrillization propensity, and yeast toxicity. *J. Mol. Biol.* 366 1510–1522. 10.1016/j.jmb.2006.12.044 17222866 PMC1868670

[B89] Walsh D. M. Selkoe D. J. (2016). A critical appraisal of the pathogenic protein spread hypothesis of neurodegeneration. *Nat. Rev. Neurosci.* 17 251–260. 10.1038/nrn.2016.13 26988744 PMC6701169

[B90] Wang L. Das U. Scott D. A. Tang Y. McLean P. J. Roy S. (2014). α-synuclein multimers cluster synaptic vesicles and attenuate recycling. *Curr. Biol.* 24 2319–2326. 10.1016/j.cub.2014.08.027 25264250 PMC4190006

[B91] Wang W. Perovic I. Chittuluru J. Kaganovich A. Nguyen L. T. T. Liao J. (2011). A soluble α-synuclein construct forms a dynamic tetramer. *Proc. Natl. Acad. Sci. U.S.A.* 108 17797–17802. 10.1073/pnas.1113260108 22006323 PMC3203798

[B92] Weinreb P. H. Zhen W. Poon A. W. Conway K. A. Lansbury P. T. Jr. (1996). NACP, a protein implicated in Alzheimer’s disease and learning, is natively unfolded. *Biochemistry* 35 13709–13715. 10.1021/bi961799n 8901511

[B93] Westphal C. H. Chandra S. S. (2013). Monomeric synucleins generate membrane curvature. *J. Biol. Chem.* 288 1829–1840. 10.1074/jbc.M112.418871 23184946 PMC3548493

[B94] Wietek J. Haralampiev I. Amoussouvi A. Herrmann A. Stöckl M. (2013). Membrane bound α-synuclein is fully embedded in the lipid bilayer while segments with higher flexibility remain. *FEBS Lett.* 587 2572–2577. 10.1016/j.febslet.2013.06.034 23831067

[B95] Winner B. Jappelli R. Maji S. K. Desplats P. A. Boyer L. Aigner S. (2011). In vivo demonstration that alpha-synuclein oligomers are toxic. *Proc. Natl. Acad. Sci. U.S.A.* 108 4194–4199. 10.1073/pnas.1100976108 21325059 PMC3053976

[B96] Wislet-Gendebien S. D’Souza C. Kawarai T. St George-Hyslop P. Westaway D. Fraser P. (2006). Cytosolic proteins regulate alpha-synuclein dissociation from presynaptic membranes. *J. Biol. Chem.* 281 32148–32155. 10.1074/jbc.M605965200 16926154

[B97] Zarbiv Y. Simhi-Haham D. Israeli E. Elhadi S. A. Grigoletto J. Sharon R. (2014). Lysine residues at the first and second KTKEGV repeats mediate α-Synuclein binding to membrane phospholipids. *Neurobiol. Dis.* 70 90–98. 10.1016/j.nbd.2014.05.031 24905915

[B98] Zarranz J. J. Alegre J. Gómez-Esteban J. C. Lezcano E. Ros R. Ampuero I. (2004). The new mutation, E46K, of alpha-synuclein causes Parkinson and Lewy body dementia. *Ann. Neurol.* 55 164–173. 10.1002/ana.10795 14755719

[B99] Zhu M. Fink A. L. (2003). Lipid binding inhibits alpha-synuclein fibril formation. *J. Biol. Chem.* 278 16873–16877. 10.1074/jbc.M210136200 12621030

